# Cost-Effectiveness Analysis of Camrelizumab *vs.* Placebo Added to Chemotherapy as First-Line Therapy for Advanced or Metastatic Esophageal Squamous Cell Carcinoma in China

**DOI:** 10.3389/fonc.2021.790373

**Published:** 2021-12-01

**Authors:** Qilin Zhang, Pan Wu, Xucheng He, Yufeng Ding, Yamin Shu

**Affiliations:** ^1^ Department of Pharmacy, Union Hospital, Tongji Medical College, Huazhong University of Science and Technology, Wuhan, China; ^2^ Department of Pharmacy, Qionglai Maternal & Child Health and Family Planning Service Center, Qionglai, China; ^3^ Department of Pharmacy, Pengzhou Second People's Hospital, Pengzhou, China; ^4^ Department of Pharmacy, Tongji Hospital, Tongji Medical College, Huazhong University of Science and Technology, Wuhan, China

**Keywords:** cost-effectiveness analysis, ESCORT-1st trial, esophageal squamous cell carcinoma, camrelizumab, first-line treatment

## Abstract

**Objective:**

The purpose of this cost-effectiveness analysis was to estimate the effects of adding camrelizumab to standard chemotherapy as the first-line treatment in patients with advanced or metastatic esophageal squamous cell carcinoma (ESCC) on health and economic outcomes in China.

**Methods:**

A Markov model was developed to simulate the clinical course of typical patients with advanced or metastatic ESCC in the ESCORT-1st trial. Weibull survival model was employed to fit the Kaplan-Meier progression-free survival and overall survival probabilities of the camrelizumab-chemotherapy and placebo-chemotherapy strategy, respectively. Quality-adjusted life-years (QALYs) and incremental cost-effectiveness ratios (ICER) were estimated over a 5-year lifetime horizon. Meanwhile, one-way and probabilistic sensitivity analyses were conducted to test the uncertainty in the model.

**Results:**

On baseline analysis, the incremental effectiveness and cost of camrelizumab-chemotherapy versus placebo-chemotherapy were 0.15 QALYs and $7,110.56, resulting in an ICER of $46,671.10/QALY, higher than the willingness-to-pay (WTP) threshold of China ($31,498.70/QALY). The results were sensitive to the utility of PFS and cost of camrelizumab.

**Conclusion:**

The findings from the present analysis suggest that the addition of camrelizumab to chemotherapy might not be cost-effective in patients with advanced or metastatic ESCC in China.

## Introduction

Esophageal cancer is the seventh most frequently diagnosed malignant cancer and ranks sixth in mortality worldwide (1). China has a high incidence of esophageal cancer, accounting for more than 50% of the global morbidity and mortality (2). Esophageal squamous cell carcinoma (ESCC) and esophageal adenocarcinoma (EAC) are the two major histological types of esophageal cancer. In China, approximately 90% of esophageal cancer patients are diagnosed with ESCC (3). Palliative chemotherapy regiments, including fluorouracil plus platinum, and paclitaxel plus platinum, are the current recommended standard first-line therapy for patients with unresectable advanced, relapsed or metastatic ESCC (4). However, the prognosis of patients with advanced ESCC is still poor. The 5-year survival rate is only 12.4% in Europe and 20.9% in China (5, 6). Therefore, new treatment options for patients with advanced or metastatic ESCC are urgently needed.

In recent years, immune checkpoint inhibitors (ICIs) have made exciting breakthroughs in cancer therapy by blocking CTLA-4 or PD-1 pathways to enhance the antitumor activity of T cells, and have also shown outstanding performance in the treatment of esophageal cancer (7, 8). Among them, KEYNOTE-181, ATTRACTION-3 and ESCORT studies focusing on advanced or metastatic ESCC patients successfully presented excellent efficacy in the second-line treatment, indicating the arrival of the era of esophageal cancer immunotherapy (7, 9, 10). The Chinese Society of Clinical Oncology (CSCO) Guidelines for the Diagnosis and Treatment of Esophageal Cancer in the 2021 edition have recommended camrelizumab combined with paclitaxel and cisplatin chemotherapy as the first-line treatment of advance or metastatic ESCC.

The world’s first phase III clinical trial of the first-line immunotherapy combined with chemotherapy for the advanced ESCC was the ESCORT-1st trial conducted in China, and we performed a cost-effectiveness analysis based on ESCORT-1st trial (11). The ESCORT-1st trial was conducted to evaluate the efficacy and adverse events of camrelizumab combined with paclitaxel and cisplatin compared with placebo combined with paclitaxel and cisplatin for the first-line treatment of advanced ESCC (11). Results demonstrated that camrelizumab combined with chemotherapy significantly prolonged median OS (mOS, 15.3 months *vs.* 12.0 months) and median PFS (mPFS, 6.9 months *vs.* 5.6 months) compared with placebo plus chemotherapy. The objective response rate was higher (ORR, 72.1% *vs.* 62.1%) and the duration of response was longer (DOR, 7.0 months *vs.* 4.6 months) with patients in the camrelizumab plus chemotherapy group. In terms of safety, the incidence of grade ≥3 treatment-related adverse events were similar in both groups (63.4% *vs.* 67.7%), with the most common grade ≥3 treatment-related adverse event being neutrophil count reduction (39.9% *vs.* 43.4%).

The statistically significant improvements in PFS and OS demonstrated the apparent benefit of camrelizumab in the treatment of advanced ESCC. However, the high cost of camrilizumab may have profound economic consequences. Hence, this study aims to assess the economics of camrelizumab plus chemotherapy for the first-line treatment of advanced or metastatic ESCC based on the ESCORT-1st trial from the perspective of the Chinese healthcare system.

## Methods

### Model Structure

**Figure 1:**
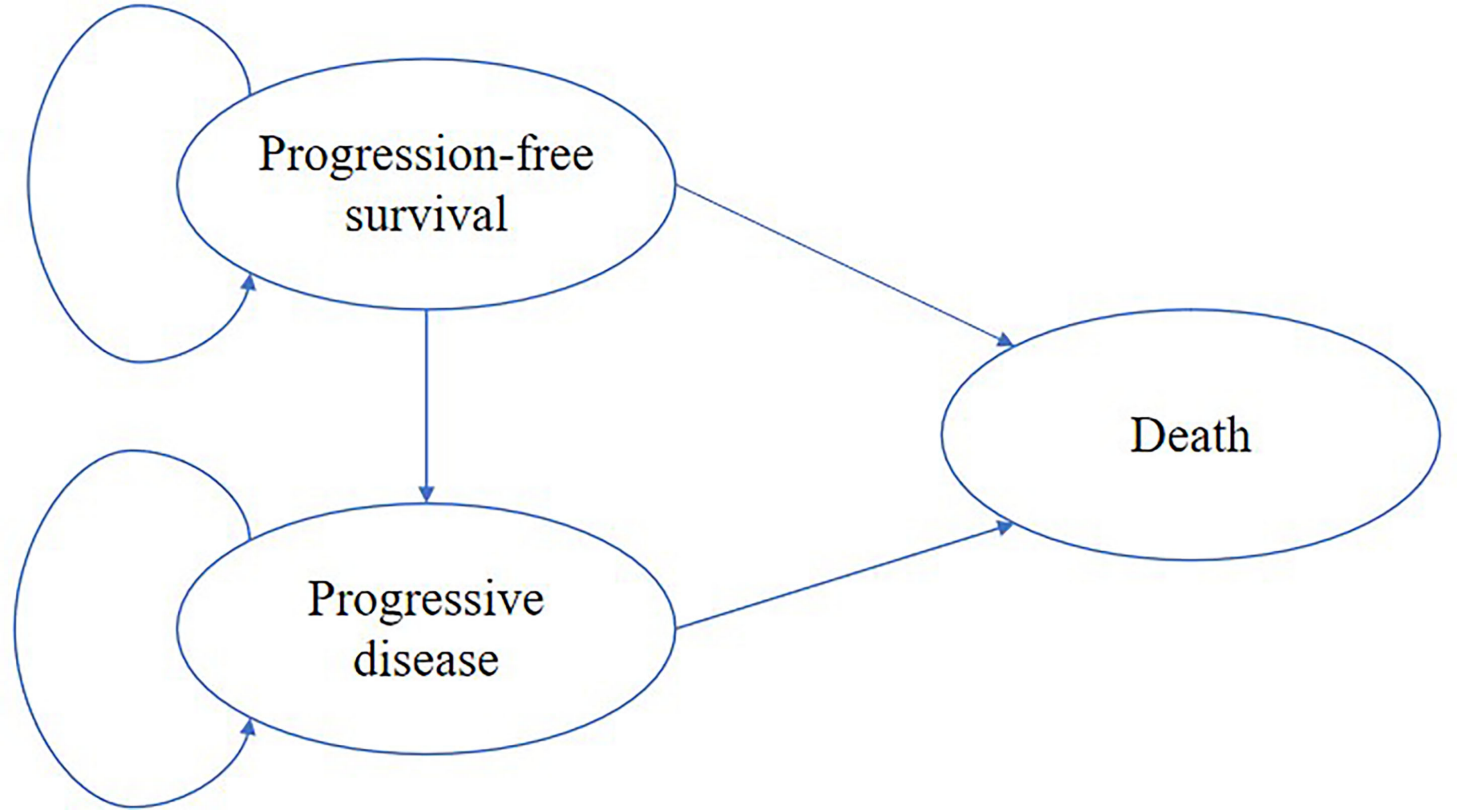
The Markov model simulated three health states: progression-free survival, progressive disease and death.

A state-transition Markov model was established to integrate clinical and economic outcomes of camrelizumab-chemotherapy versus placebo-chemotherapy as first-line therapy for patients with advanced or metastatic ESCC in China. The model comprised three mutually exclusive health states: progression-free survival (PFS), progressive disease (PD) and death (**Figure 1**). The initial health state for all patients was PFS and patients either remained in their assigned health state or progressed to a new health state during each Markov cycle (12). The tracked time horizon of the model was 5 years and the Markov cycle in the model was 1 month. The primary outcomes were quality-adjusted life-years (QALYs) and cost in the study. The future costs and benefits were discounted using a 3% annual discount rate according to the WHO guidelines for pharmacoeconomic evaluations (13). All costs had been adjusted to 2020 prices according to the local Consumer Price Index and were presented in US dollars ($1 = ¥6.9). A cost-effectiveness analysis was conducted to evaluate the outcomes of the two strategies and presented as incremental cost-effectiveness ratios (ICERs). The formula used to calculate the ICER as following: ICER = [Cost (camrelizumab)-Cost (placebo)]/[QALY (camrelizumab)-QALY (placebo)]. We used 3×the per capita gross domestic product (GDP) of China in 2020 ($31,498.70) as the willingness-to-pay (WTP) threshold according to the WHO recommendations. Model development and outcomes analysis were performed in the TreeAge Pro 2019 software (Williamstown, MA, USA) and R software (version 4.0.5, Vienna, Austria). This economic analysis was based on a randomized clinical trial and an experimental model and did not require approval from an institutional review board or ethics committee.

### Clinical Data

The clinical efficacy and safety data were based on the patients in the ESCORT-1st trial, a randomized, double-blind, placebo-controlled, multicenter, phase 3 trial enrolled patients from 60 hospitals in China (11). Patients were eligible if they conformed to the following conditions: 1. 18-75 years old and had adequate organ function; 2. cytologically or histologically confirmed ESCC; 3. unresectable, locally advanced, or recurrent disease that precluded esophagectomy or definitive chemoradiation, or distant metastatic disease; 4. received no previous systemic therapy (patients who had progressed ≥6 months after definitive chemoradiation were eligible); 5. an Eastern Cooperative Oncology Group performance status score of 0 or 1, and had at least 1 measurable lesion according to the Response Evaluation Criteria in Solid Tumors (RECIST) version 1.1; 6. a life expectancy of at least 12 weeks. Eligible patients were randomly assigned in a 1:1 ratio to either the camrelizumab-chemotherapy group (n = 298) or the placebo-chemotherapy group (n = 298). Camrelizumab (200 mg) or placebo were given every 3 weeks until disease progression or unacceptable toxicity. Paclitaxel (175 mg/m^2^) and cisplatin (75 mg/m^2^) were given every 3 weeks up to 6 cycles after randomization. The median OS was 15.3 months (95% CI:12.8-17.3) in the camrelizumab-chemotherapy group and 12.0 months (95% CI: 11.0-13.3) in the placebo-chemotherapy group. The median PFS was 6.9 months (95% CI: 5.8-7.4) in the camrelizumab-chemotherapy group and 5.6 months (95% CI:5.5-5.7) in the placebo-chemotherapy group.

**Figure 2:**
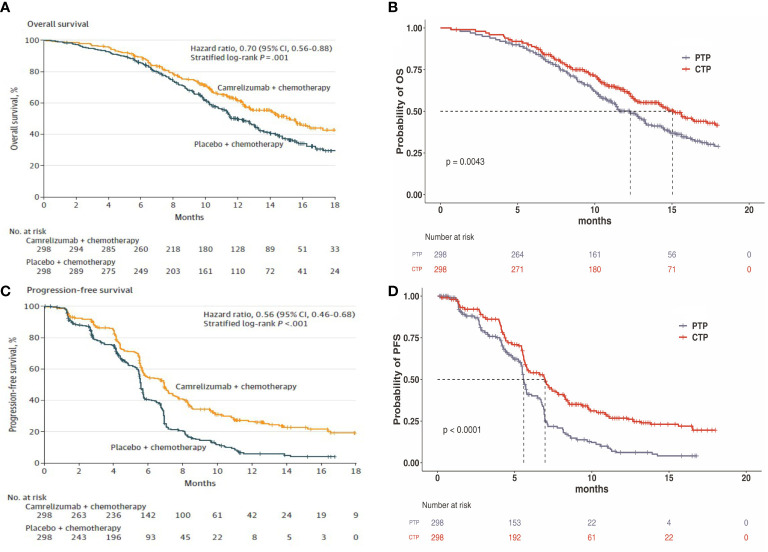
**(A)** Kaplan–Meier curve of the overall survival from the ESCORT-1st trial. **(B)** Simulate overall survival curve for the CTP group and the PTP group. **(C)** Kaplan-Meier curve of progression-free survival from the ESCORT-1st trial. **(D)** Simulate progression-free survival curve for the CTP group and the PTP group. CTP, camrelizumab-chemotherapy; PTP, placebo-chemotherapy; OS, overall survival; PFS, progression-free survival.

Transition probabilities between the different health states were estimated from Kaplan-Meier survival curves which obtained from the ESCORT-1st trial. As individual patient data were not available, the Kaplan-Meier curves of PFS and OS for the two groups were read by GetData Graph Digitizer software (Version 2.26), which digitized data points from an image file. To extrapolate the probability of survival beyond the observation period, the Weibull distribution was fitted to the data for PFS and OS curves using R statistical software (version 4.0.5, Vienna, Austria). The estimated scale (λ) and shape (γ) parameters, standard error, and 95% confidence interval were presented in **Table 1**. Formula S(t)=exp(-λt^γ^) was used to calculate the survival probability at time t and we used formula P(t)=1-exp[λ(t-1)^γ^-λt^γ^] to estimate the transition probability at a given cycle t (14, 15). The transition probability from PFS to death state is derived from the natural death rate of Chinese population in 2020 (0.707%) (16). The survival curve simulation results were shown in **Figure 2**.

**Table 1:** Weibull parameters of model estimated for progression-free and overall survival curves.

Group		Parameter	Mean	SE	95% CI	
					Low	Up
CTP	PFS	scale (λ) shape (γ)	0.035843 1.440454	0.007191 0.082920	0.024190 1.286766	0.053110 1.612498
	OS	scale (λ) shape (γ)	0.005274 1.798021	0.001911 0.135765	0.002593 1.550680	0.010729 2.084815
PTP	PFS	scale (λ) shape (γ)	0.030222 1.824045	0.005986 0.092109	0.020499 1.652161	0.044558 2.013811
	OS	scale (λ) shape (γ)	0.006991 1.818036	0.002212 0.120060	0.003760 1.597315	0.013000 2.069258

CTP, camrelizumab-chemotherapy; PTP, placebo-chemotherapy; PFS, progression-free survival; OS, overall survival; SE, standard error; 95% CI, 95% confidence interval.

### Costs and Utilities

Costs were estimated from the perspective of the Chinese healthcare system. Only direct medical costs, including the costs of camrelizumab and chemotherapy, laboratory tests and radiological examinations, management of treatment-related grade 3-4 serious adverse events (SAEs), best supportive care (BSC), cost of salvage therapy per cycle, routine follow-up and terminal care in end-of-life, were included in the model (**Table 2**). To estimate the dosage of chemotherapeutic agents (17), it was assumed that a typical patient weighed 65 kg and had a height of 1.64 m, resulting in a body surface area (BSA) of 1.72 m^2^. The costs related to SAEs were calculated by multiplying the incidence of the SAEs by the costs of managing the SAEs per event. The most common adverse events, including anemia, white blood cell count decreased, neutrophil count decreased, and the incidence rates of adverse events that occurred with two groups were obtained from the ESCORT-1st trial (11). Once the disease progressed, salvage chemotherapy and best supportive care were prescribed. All costs were derived from local hospitals or previously published studies (17–19). As no data on quality of life were estimated in the ESCORT-1st trial, the utility scores of PFS and survival after progression were obtained from the literature (19). Furthermore, terminal cost and a half-cycle correction were implemented, according to the TreeAge Pro 2019 manual.

**Table 2:** Model economic parameters and the range of the sensitivity analysis.

Variables	Base Case (Rang)	Distribution	Source
Costs ($)			
Camrelizumab (200 mg)	424.35 (339.40-509.10)	Triangle	Local charge
Paclitaxel (100 mg)	108.26 (86.61-129.91)	Triangle	Local charge
Cisplatin (100 mg)	10.97 (8.78-13.16)	Triangle	Local charge
Routine follow-up cost per cycle	73.57 (58.86-88.28)	Triangle	(17)
Cost of laboratory tests and radiological examinations	356.60 (285.28-427.92)	Triangle	(17)
Cost of salvage therapy per cycle	638.43 (510.74-766.12)	Triangle	Local charge
Cost of supportive care per cycle	167.29 (133.83-200.75)	Triangle	(17)
Cost of terminal care in end-of-life	1,460.30 (1,168.24-1,752.36)	Triangle	(18)
Costs of serious adverse events ($)			
Anemia	508.2 (381.2-635.3)	Triangle	(17)
White blood cell count decreased	466.00 (372.80-559.20)	Triangle	(17)
Neutrophil count decreased	534.40 (427.52-641.28)	Triangle	(19)
Risks of serious adverse events in CTP group (grade 3 or 4) %			
Anemia	17.40 (13.92-20.88)	Beta	(11)
White blood cell count decreased	24.20 (19.36-29.04)	Beta	(11)
Neutrophil count decreased	39.90 (31.92-47.88)	Beta	(11)
Risks of serious adverse events in PTP group (grade 3 or 4) %			
Anemia	13.50 (10.80-16.20)	Beta	(11)
White blood cell count decreased	26.60 (21.28-31.92)	Beta	(11)
Neutrophil count decreased	43.40 (34.72-52.08)	Beta	(11)
Utility value			
PFS	0.68 (0.54-0.82)	Beta	(19)
PD	0.42 (0.34-0.50)	Beta	(19)
Body surface area (m^2^)	1.72 (1.38-2.06)	Triangle	(17)
Discount rate (%)	3 (0–8)	Fixed in PSA	(13)

CTP, camrelizumab-chemotherapy; PTP, placebo-chemotherapy; PFS, progression-free survival; PD, progressive disease; PSA, probabilistic sensitivity analyses.

### Sensitivity Analyses

To assess the impact of uncertainty in model inputs on the outcomes, one-way and probabilistic sensitivity analyses (PSA) were performed in this research. In the one-way sensitivity analysis, relevant parameters were changed one-by-one to their respective upper and lower boundaries, with a range of ± 20% of the base case value, in order to identify the parameters that most significantly influenced the economic outcomes. The result of the one-way sensitivity analysis was presented in a Tornado diagram. The PSA was performed to assess the effects of uncertainty in all model parameters simultaneously. The model was run 1000 times, in which the parameters were changed with a specific pattern of distribution (triangle distribution for costs, beta distribution for the probability parameters and utilities). The results of the PSA were presented as cost-effectiveness acceptability curve and probabilistic scatter plot, to estimate the WTP threshold for an incremental unit of effectiveness.

## Results

### Base Case Analysis

The base case analysis showed that over 5-year time horizon, camrelizumab-chemotherapy group gained 0.79 QALYs at a cost of $20,460.60. In the placebo-chemotherapy group, the effectiveness was 0.64 QALYs while the cost was $13,350.04. Compared with placebo-chemotherapy, the mean incremental effect and cost were 0.15 QALYs and $7,110.56 for the camrelizumab-chemotherapy group. The ICER for camrelizumab-chemotherapy versus placebo-chemotherapy was $46,671.10/QALY (**Table 3**). At the Chinese cost-effectiveness WTP threshold of $31,498.70/QALY, camrelizumab-chemotherapy was not a cost-effective treatment strategy compared with placebo-chemotherapy.

**Table 3:** The cost and outcome results of the cost-effectiveness analysis.

Parameters	CTP group	PTP group
Costs ($)		
PFS state	13,518.28	6,180.97
PD state	6,942.32	7,169.07
Total Cost	20,460.60	13,350.04
Incremental costs ($)	7,110.56	/
Effectiveness (QALYs)		
PFS state	0.55	0.39
PD state	0.24	0.25
Total effectiveness	0.79	0.64
Incremental effectiveness (QALYs)	0.15	/
ICER ($/QALY)	46,671.10	/

CTP, camrelizumab-chemotherapy; PTP, placebo-chemotherapy; PFS, progression-free survival; PD, progressive disease; QALYs, Quality-adjusted life-years; ICER, incremental cost-effectiveness ratios.

### Sensitivity Analyses

**Figure 3:**
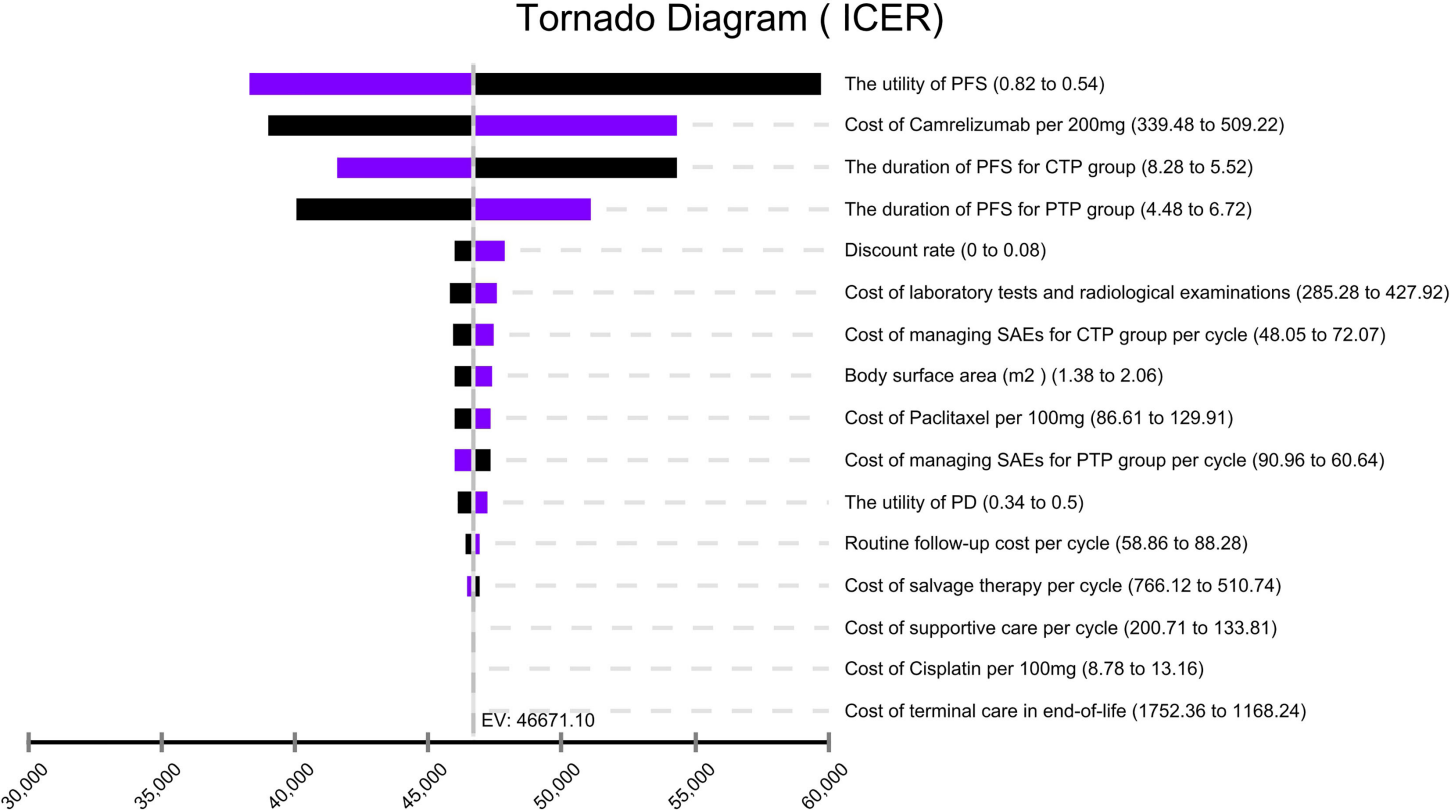
Tornado diagram of one-way sensitivity analysis. It summarized the results of one-way sensitivity analysis, which listed influential parameters in descending order according to their effect on the ICER over the variation of each parameter value. ICER, incremental cost-effectiveness ratios; CTP, camrelizumab-chemotherapy; PTP, placebo-chemotherapy; PFS, progression-free survival; PD, progressive disease; SAEs, serious adverse events.

**Figure 4:**
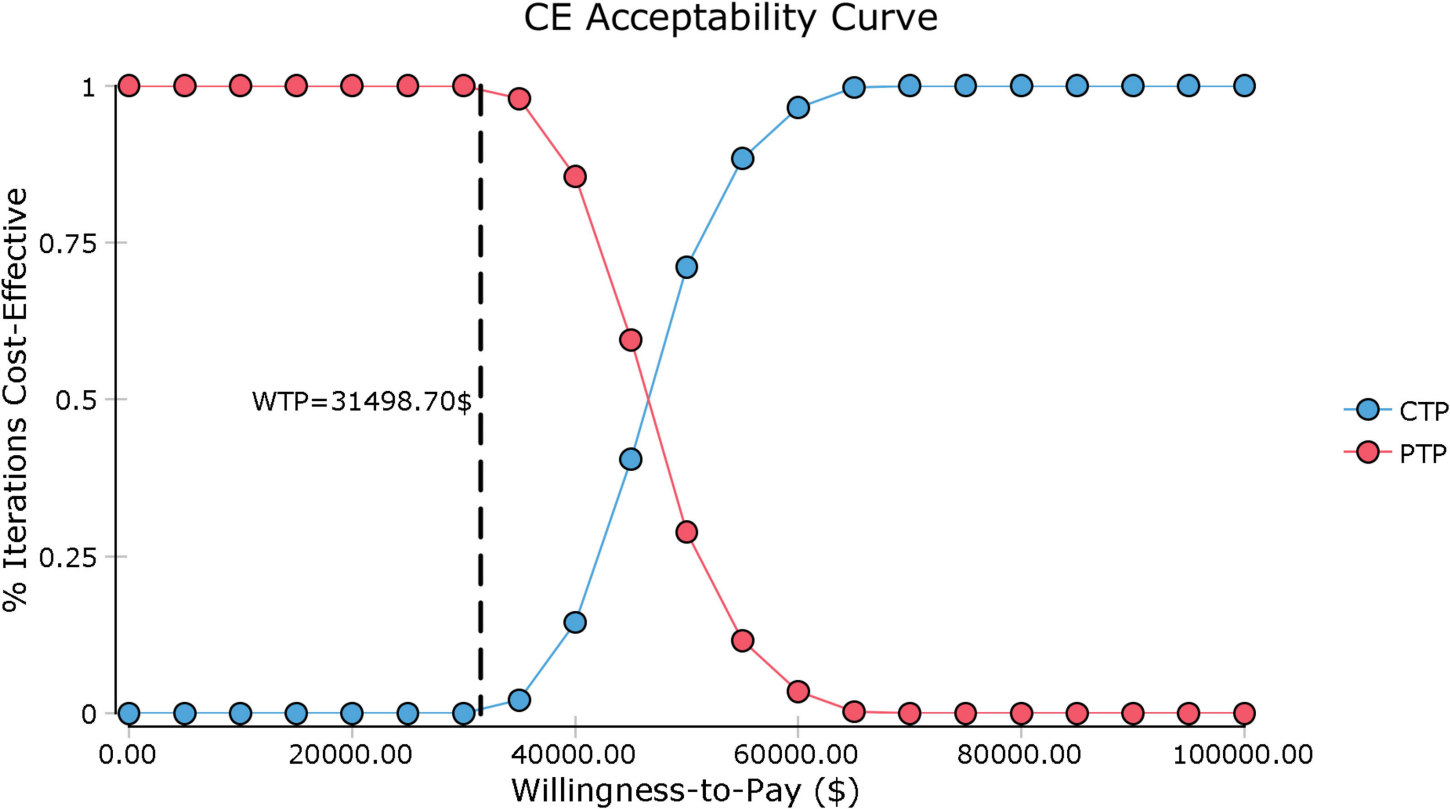
Cost-effectiveness acceptability curve. CE, cost-effectiveness; CTP, camrelizumab-chemotherapy; PTP, placebo-chemotherapy; WTP, willingness-to-pay.

**Figure 5:**
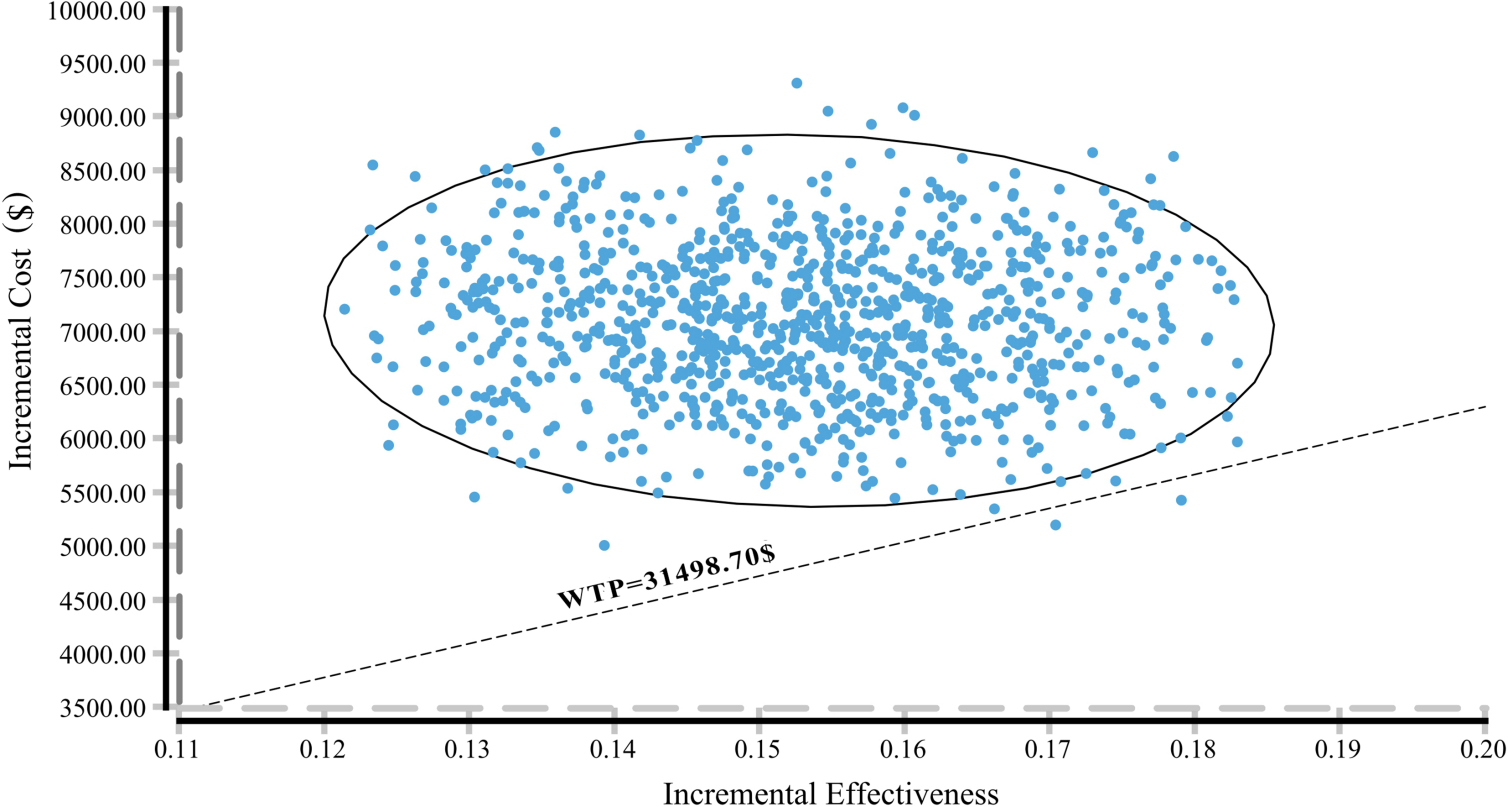
A probabilistic scatter plot of the ICER between the CTP and PTP group. Each dot represents the ICER for 1 simulation. An ellipse means 95% confidence interval. Dots that are located below the ICER threshold represent cost-effective simulations. CTP, camrelizumab-chemotherapy; PTP, placebo-chemotherapy; WTP, willingness-to-pay, ICER, incremental cost-effectiveness ratios.

In the tornado diagram of one-way sensitivity analysis (**Figure 3**), the most influential variables were the utility of PFS and the cost of camrelizumab per 200 mg. However, altering these parameters could not yield substantial changes in the ICER, $38,293.88-$59,739.88/QALY and $38,999.36-$54,342.85/QALY, respectively. Other parameters influencing the model were the duration of PFS, discount rate, cost of laboratory tests and radiological examinations, cost of managing SAEs, body surface area, cost of paclitaxel per 100 mg. Changes in parameters, the utility of PD, routine follow-up cost per cycle, and the costs of salvage therapy per cycle, supportive care per cycle, cisplatin per 100 mg, terminal care in end-of-life had a mild impact on economic outcomes. Nevertheless, none of the variables could reduce the ICERs below the thresholds. The cost-effectiveness acceptability curve and probabilistic scatter plot were shown in **Figures 4**, **5**. Regardless of the scenarios, the camrelizumab-chemotherapy group was cost-effective in approximately less than 1% of the simulations compared with placebo-chemotherapy group, with a cost-effectiveness threshold of $31,498.70 in China.

## Discussion

ESCC is one of the most commonly malignant gastrointestinal tumors globally. Palliative chemotherapy as the first-line treatment for advanced/refractory ESCC, which not only had limited survival benefits, but also had poor prognosis and relatively high adverse reactions. ICIs significantly improved survival and quality of life in a range of malignancies by inhibiting the CTLA-4 or PD-1 pathway (20, 21). On September 14, 2021, the ESCORT-1st trial, the world’s first phase III clinical trial using immunotherapy combined with chemotherapy as first-line treatment for advanced ESCC, was completed at more than 60 hospitals in China and published in ⟪JAMA⟫, comparing the efficacy and safety of camrelizumab combined with paclitaxel and cisplatin versus placebo combined with paclitaxel and cisplatin (11). As compared to standard chemotherapy, camrilizumab-chemotherapy significantly prolonged patients’ median OS and median PFS, reducing the risk of death by 30% and the risk of disease progression by 44%. It has achieved the longest OS (15.3 months) and the highest response rate (72.1%) in the field of first-line treatment for esophageal cancer, which provided a novel first-line treatment option for patients with ESCC.

However, the price of ICIs is usually high, which may significantly increase the healthcare expenditures. Hence, it is important to evaluate the effect of ICIs from the perspective of pharmacoeconomics. In choosing a phase III trial for cost-effectiveness analysis, ESCORT-1st trial was the best choice. In this study, our analysis showed that the ICER of camrelizumab-chemotherapy for first-line treatment of advanced ESCC in China was $46,671.10/QALY and the WTP threshold was $31,498.70/QALY, revealing that camrelizumab-chemotherapy strategy may not be a cost-effective treatment option compared with chemotherapy.

In the one-way sensitivity analysis, the utility of PFS and the cost of camrelizumab per 200 mg had the highest impacts on the ICER. Probabilistic sensitivity analysis of 1000 Monte Carlo simulations was adopted to alter the cost of camrelizumab. Only about 1% of simulations in the camrelizumab-chemotherapy group are cost-effective at the WTP threshold ($31,498.70/QALY). The ICER ($31,362.86/QALY) approached the WTP threshold with cost-effectiveness when the price of camrilizumab was reduced to $255/200 mg in China. However, different regions have different cost-effectiveness WTP threshold value. The ICER in the camrelizumab-chemotherapy group was higher than the threshold recommended by wealthier developed countries, such as £20,000-30,000 per QALY proposed by the UK’s National Institute for Health and Care Excellence (NICE) (22). Assuming that the prices of camrelizumab and chemotherapy remain constant, camrelizumab-chemotherapy may not be cost-effective as a first-line treatment for patients with advanced or metastatic ESCC in other countries as well. Particularly, camrelizumab-chemotherapy strategy might be the optimal alternative option in developed cities and provinces of China, such as Beijing (WTP = $72,886.96/QALY), Shanghai (WTP = $69,297.83/QALY), Jiangsu (WTP = $55,341.30/QALY), Fujian (WTP = $48,046.09/QALY) and Zhejiang (WTP = $48,021.74/QALY), which had over 50% chance to be cost-effective. In addition, the utility of PFS had a higher impact on the model outcomes, but even if the utility of PFS varied from 0.42 to 1, the ICER ranged from $78,606.77/QALY to $31,513.53/QALY, which was still higher than the WTP.

Currently, pharmacoeconomic studies on ESCC were limited, with only 10 articles been searched in PubMed, and most of them focused on screening, surgical techniques or chemoradiation (23–25). There were only two economic analyses of immunotherapy for ESCC. A recent study was the cost-effectiveness analysis of nivolumab in the second-line treatment for advanced ESCC. Their study included 419 advanced ESCC patients and showed an ICER of $136,709.35/QALY for nivolumab versus chemotherapy at a $29,306.43/QALY WTP threshold (17). From the perspective of Chinese society, nivolumab is not a cost-effective treatment option compared with chemotherapy, which were basically consistent with our results. Another study compared the economics of camrelizumab versus chemotherapy as second-line therapy for advanced ESCC (26). The study included 457 advanced ESCC patients at 43 hospitals, demonstrating that camrelizumab had higher QALYs (0.782 *vs.* 0.499) and higher cost ($31,537 *vs.* $6,998) than chemotherapy. The ICER of camrilizumab versus chemotherapy was $86,745/QALY, which was far above the WTP threshold ($30,094/QALY gained). Therefore, camrelizumab is not cost-effective in China compared with chemotherapy as second-line treatment for advanced or metastatic ESCC. Generally, the prices of PD-1 inhibitors in China are higher than those of conventional chemotherapy (17, 26). Based on previous studies and our results, it is demonstrated that camrelizumab was not cost-effective compared with chemotherapy, whether it is first-line treatment or second-line treatment for advanced or metastatic ESCC in China. Consequently, from the perspective of policy, the price of camrelizumab needs to be reduced to reduce the financial burden on the healthcare system and provide more access to Chinese patients.

In our study, higher QALYs (0.79 *vs.* 0.64) are obtained in camrelizumab- chemotherapy as first-line treatment for advanced or metastatic ESCC compared with chemotherapy. The ICER is $46,671.10/QALY. Although it is not economical, the ICER value is lower compared with second-line treatment. One possible reason is that the cost of camrelizumab per 200 mg has fallen from $2,802 in 2020 to $424 in 2021 (26). The second may be the effect of camrelizumab as first-line treatment is better than second-line treatment and it also could be the different utility values of PFS and PD in different studies. In recent years, China has formulated a series of preferential policies for antitumor drugs. In addition, with the continuous improvement of national medical insurance policies and the unique price advantage brought by volume-based procurement, the prices of PD-1 inhibitors may be further reduced, and this treatment could help ESCC patients obtain a first-line treatment that is safer and has a longer overall survival rate than traditional chemotherapy.

This study has several advantages. First, this is the first cost-effectiveness analysis of camrelizumab combine with chemotherapy as first-line treatment for advanced or metastatic ESCC in China and the world. In addition, it is also the largest ESCC immunotherapy trial with the largest sample size, longest overall survival and highest response rate among first-line therapies. Therefore, the results of this analysis could be taken into consideration by the National Healthcare Security Administration in its annual price negotiations. Our study inevitably had some limitations that warrant discussion. First, due to lack of long-term (>5 years) survival data, we used a two-parameter Weibull survival model to extrapolate the tails of survival beyond the follow-up time horizon, which may not accurately reflect the real world condition (27). The current cost-effective analysis must be updated when long-term survival data are reported. Second, we assumed patients received paclitaxel after disease progression, which may not reflect the current Chinese clinical practice situation precisely because patients might switch to subsequent therapy upon the further progression. However, the result of the sensitivity analysis supported that the costs associated with disease progression did not have an important impact on economic outcomes. Third, we only considered the most common grade 3/4 SAEs in the model. We hypothesized that low-probability adverse events would not change the final conclusions of the study, and the sensitivity analysis showed that the result was not sensitive to SAEs-related parameters. Fourth, although all patients in the ESCORT-1st trial were from China, the utility values in this study were derived from western countries, which might lead to bias in the model outcomes. Finally, due to the strict eligible conditions of clinical trials and the unbalanced economic development in various regions of China, the applicability of this study may be limited. Despite these limitations, this study might be a valuable reference for decision makers about camrelizumab as a first-line treatment for advanced or metastatic ESCC in China.

## Conclusion

In conclusion, camrelizumab combined with chemotherapy treatment is unlikely to be considered cost-effective as compared to conventional chemotherapy as a first-line treatment for advanced or metastatic ESCC from the perspective of the Chinese healthcare system. However, if the price is reduced, camrelizumab may be a cost-effective treatment option. Our results are potentially helpful to healthcare systems decision-making, but real-world studies are further needed to verify the efficacy, safety and economics of these regimens for first-line therapy of ESCC.

## Data Availability Statement

The raw data supporting the conclusions of this article will be made available by the authors, without undue reservation.

## Author Contributions

Conceptualization, QZ, YD, and YS. Data curation, YS. Formal analysis, QZ, YD, and YS. Funding acquisition, QZ. Methodology, PW, XH, and YS. Project administration, QZ and YS. Software, YS. Supervision, QZ, YD, and YS. Validation, QZ. Writing – original draft, QZ, PW, XH, YD, and YS. Writing – review & editing, QZ, YD, and YS. All authors contributed to the article and approved the submitted version.

## Funding

This work was supported by the National Natural Science Foundation of China (No. 82104476) and National Key R&D Program of China (No. 2017YFC0909900).

## Conflict of Interest

The authors declare that the research was conducted in the absence of any commercial or financial relationships that could be construed as a potential conflict of interest.

## Publisher’s Note

All claims expressed in this article are solely those of the authors and do not necessarily represent those of their affiliated organizations, or those of the publisher, the editors and the reviewers. Any product that may be evaluated in this article, or claim that may be made by its manufacturer, is not guaranteed or endorsed by the publisher.
